# Microbial Methylation of Iodide in Unconfined Aquifer Sediments at the Hanford Site, USA

**DOI:** 10.3389/fmicb.2019.02460

**Published:** 2019-10-24

**Authors:** Christopher E. Bagwell, Lirong Zhong, Jacqueline R. Wells, Alexandre V. Mitroshkov, Nikolla P. Qafoku

**Affiliations:** Pacific Northwest National Laboratory, Earth Systems Science Division, Richland, WA, United States

**Keywords:** methylation, volatilization, radioiodine, vadose zone, iodine cycling

## Abstract

Incomplete knowledge of environmental transformation reactions limits our ability to accurately inventory and predictably model the fate of radioiodine. The most prevalent chemical species of iodine include iodate (IO_3_^−^), iodide (I^−^), and organo-iodine. The emission of gaseous species could be a loss or flux term but these processes have not previously been investigated at radioiodine-impacted sites. We examined iodide methylation and volatilization for Hanford Site sediments from three different locations under native and organic substrate amended conditions at three iodide concentrations. Aqueous and gaseous sampling revealed methyl-iodide to be the only iodinated compound produced under biotic conditions. No abiotic transformations of iodide were measured. Methyl-iodide was produced by 52 out of 54 microcosms, regardless of prior exposure to iodine contamination or the experimental concentration. Interestingly, iodide volatilization activity was consistently higher under native (oligotrophic) Hanford sediment conditions. Carbon and nutrients were not only unnecessary for microbial activation, but supplementation resulted in >three-fold reduction in methyl-iodide formation. This investigation not only demonstrates the potential for iodine volatilization in deep, oligotrophic subsurface sediments at a nuclear waste site, but also emphasizes an important role for biotic methylation pathways to the long-term management and monitoring of radioiodine in the environment.

## Introduction

Spent nuclear fuel reprocessing facilities are responsible for significant releases of ^129^I, a long-lived radioisotope of iodine, to the environment (Muramatsu and Ohmomo, 1986; Handl et al., 1993; Rao et al., 2002). In fact, discharges stemming from fuel reprocessing far exceed those from weapons testing and accidental releases combined (Hou and Hou, 2012). Major contributors to the global inventory of ^129^I include the La Hague and Sellafield facilities in Europe, where an estimated 5,600 and 440 kg of ^129^I have been released to the sea and atmosphere, respectively, as of 2008. Past nuclear production and fuel processing in the United States at the Department of Energy Hanford Site (1944 – 1987) released approximately 266 kg of ^129^I to the air and subsurface, resulting in over 1,500 acres of contaminated soil and groundwater plumes exceeding 50 km^2^ at concentrations ranging from 40 to 4 pCi/L (Zhang et al., 2013; Kaplan et al., 2014). Radioiodine has the lowest drinking water standard in the U.S. Federal Registry at 1 pCi/L, and thus will be a major environmental cleanup challenge for many locations that are directly affected by anthropogenic releases.

At the Hanford Site (located in southeastern Washington, USA) large volumes of processing waste, including stable (^127^I) and radio-isotopes (^129^I, ^131^I) of iodine among other contaminants, were discharged to the ground through unlined ponds and infiltration units, resulting in widespread contamination of the deep vadose zone and saturated aquifer sediments (Gee et al., 2007; Kaplan et al., 2014). There are three major chemical species of iodine in the environment: iodate (IO_3_^−^), iodide (I^−^), and organo-iodine. Iodine speciation is controlled by complex physico-chemical factors and microbial activities that strongly influence chemical behavior and mobility (reviewed by Kaplan et al., 2014 and Yeager et al., 2017). For example, high levels of organic matter in the soils and sediments at Sellafield and Chernobyl (Hou et al., 2003) as well as the Savannah River Site (Xu et al., 2011) effectively sequester iodine species on solid phases in these systems (Schmitz and Aumann, 1995; Santschi and Schwehr, 2004; Xu et al., 2011; Li et al., 2017; Hao et al., 2018). By contrast, sediments at the Hanford Site are naturally low in organic carbon (< 1 mg/L); thus, the minerology, specifically iron/ manganese oxides and calcium carbonates, as well as pH play a larger role in iodine speciation and sorption to subsurface sediments (Hu et al., 2005; Kerisit et al., 2018; Lawter et al., 2018). Site-specific conditions have an important and differentiating impact on the biogeochemical cycling and fate of iodine in environmental systems (e.g., Zhang et al., 2013), but the relative contribution of volatilization pathways at radioiodine-contaminated sites has not been investigated.

Studies of iodine speciation and mass flux at contaminated sites generally do not include quantitative metrics for microbial catalyzed formation of volatile I species. Iodine cycling has been most intensively studied in marine and coastal systems where iodine is naturally abundant (Whitehead, 1984; Fuge and Johnson, 1986). Diverse algae, fungi, and bacteria have the innate capacity for volatilizing iodide as an organo-I compound(s), CH_3_I being the predominant product (Harper, 1985; Amachi et al., 2001, 2003; Manley, 2002; Redeker et al., 2004; Amachi, 2008; Duborská et al., 2017). Annual production estimates of organo-I by marine macroalgae and phytoplankton are on the order of 10^9–10^ g CH_3_I/year (Moore and Groszko, 1999). Iodine volatilization could have important implications for the potential flux of ^129^I to the atmosphere for sites within close proximity to coastal environments, like the Sellafield nuclear facility and the Fukushima-Daiichi nuclear power complex in Japan. Less relevant information is available for inland, terrestrial systems but a number of positive indicators imply that the sediments in the capillary fringe at the Hanford Site could be conducive to iodine methylation. First, reactive Fe and Mn mineral fractions in unsaturated Hanford sediments have been shown to affect the distribution of U, Tc, and Cr valence species (Fredrickson et al., 2004; Zachara et al., 2007); thus, it is conceivable that this reduction capacity would transform iodate to iodide (Hu et al., 2005; Xu et al., 2015). Second, studies have shown that iodide methylation persists under oligotrophic conditions, like those at Hanford; and third, a positive correlation has been established between methylation activity and environmental concentrations of iodine (stable I^127^). The potential for iodine volatilization at nuclear impacted sites with ^129^I contamination has not been systematically evaluated but this pathway could have important implications for the long-term management of legacy waste sites.

The goal of this investigation was to measure the capacity of ^129^I impacted Hanford sediments to methylate and volatilize iodide as a first step in evaluating whether a volatilization pathway could be a controlling factor on the fate and distribution of iodine in subsurface sediments. This study establishes a new level of understanding of subsurface microbial features and activities that can contribute to the flux of iodine between the vadose zone (gas phase) and groundwater (liquid phase).

## Experimental

### Hanford Sediments

Microcosms were prepared from sediments taken from two Hanford subsurface cores that were drilled in 2017 and held in 4°C storage for preservation (Table 1). Contaminated sediments originated from a core collected in the UP-1 radioiodine plume located on the central plateau region of the Hanford Site. Specific depth intervals were sampled for low radioiodine sediments (275′ depth; < 3.0 μg/L ^129^I) and high-radioiodine sediments (317′ depth, 10 μg/L ^129^I). Background sediments (ZP-1) were cored outside the perimeter of the contaminant plume; sediments from 198′ depth were used for microcosms. Stable iodine (^127^I) was discharged along with radioiodine (^129^I) at the Hanford Site; thus, impacted sediments do contain both isotopes. It is generally assumed that concentrations of ^127^I can exceed ^129^I by 2–3 orders of magnitude.

**Table 1:** Characteristics features of ZP-1 and UP-1 sediments.

Location	Depth (ft)	16SrRNA gene copies g^−1^ sediment (± SD)	Water table (ft)
ZP-1, No ^129^I	198	5.37 × 10^5^ (± 0.6)	240
UP-1, 1 μg/L ^129^I	275	1.27 × 10^6^ (± 0.05)	304
UP-1, 10 μg/L ^129^I	317	2.79 × 10^5^ (± 0.1)	304

### Bacterial Enumeration by QPCR

Genomic DNA was extracted from Hanford sediments (0.25 g) using the DNeasy PowerLyzer PowerSoil^®^ DNA Isolation Kit per the manufacturer’s instructions. DNA extracts were pooled and concentrated by ethanol precipitation in high salt with GlycoBlue™ Coprecipitant (50 μg/ml; Ambion). DNA yields were quantified using the NanoDrop 1000 spectrophotometer (Thermo Scientific). QPCR assays were performed in triplicate on a Bio-Rad CFX96 Real-Time PCR Detection System using the SsoAdvanced™ Universal SYBR^®^ Green Supermix (Bio-Rad) as instructed by the manufacturer and universal 16S rRNA gene primers spanning the V2-V3 hypervariable region (Fuks et al., 2018). Amplification specificity was assessed by melt curve analysis. Cell equivalents were calculated from calibration curves using pure genomic DNA from *Desulfovibrio vulgaris* (DSM-644) and *Geobacter metallireducens* (DSM-7210) as described by He et al. (2003).

### Experimental Microcosms

Hanford sediments (5 g) were aseptically weighted into sterile 160 ml serum bottles and combined with filter-sterilized synthetic groundwater to simulate saturated (1:4 ratio of sediment to synthetic groundwater). To measure the effect of organic substrate amendment on iodine volatilization, glucose and yeast extract were added to microcosms at 1 mM (final concentration) and 20 g/L, respectively. Abiotic controls included sediments that were “heat killed” at 100°C for 60 min, and native controls constituted sediments incubated “as-is” without the addition of any synthetic groundwater. Microcosms were spiked with a potassium iodide stock solution to achieve a final added I^−^ concentration of 0, 150, 200, and 250 μg/L. All experimental treatments were performed in triplicate. Resazurin was added to all bottles (1 mg/L final concentration) to monitor relative changes to the redox potential as a function of treatment conditions and incubation time. Bottles were fitted with a sterile butyl rubber stopper, crimp sealed, and incubated in the dark at 28°C for 40 days.

### Gas Phase Analysis

Headspace gas analysis was performed using an Agilent GC-MS system in selected ion monitoring mode (SIM). Iodinated organic standards were prepared with analytical grade chemicals (iodomethane, iodoethane, and 1-iodopropane) at 2, 10, 50, 100, and 200 μg/L. Controls and experimental microcosms were heated at 70°C for 30 min prior to headspace analysis. Samples were collected using a gas-tight syringe and separated on a GasPro 60 m PORAPLOT column held initially at 40°C, for 4 min and then to 220°C at a ramp rate of 20°C/min and held for 5 min.

### Aqueous Phase Analysis

Solvent extracted aqueous phase samples were analyzed by GC-MS/MS (Agilent 7890B GC, Agilent 7000C triple quad MS/MS) in SIM mode. Iodinated organic standards were prepared with analytical grade chemicals (Iodoethane, 1-Iodopropane, 2-Iodopropane, 2-Iodobutane, 2-Iodo-2-methylpropane) at a range of concentrations. Post-incubation, synthetic groundwater was separated from Hanford sediments in the controls and experimental microcosms by centrifugation (1,000 g for 20 min) and extracted with methylene chloride (2 ml) for 12 h at room temperature by continuous mixing. The methylene chloride phases were analyzed on a HP-5MS 50 m × 200 μm × 0.33 μm GC column operated at an initial temperature of 50°C for 3 min, ramped to 250°C at 10°C/min and held for 4 min, and finally ramped to 300°C at 20°C/min to 300°C and held for 0.5 min.

## Results and Discussion

The purpose of this study was to evaluate the potential for iodide volatilization at a former nuclear production and processing facility where ^129^I contamination persists in subsurface sediments and groundwater. The microbiological response to incubation in synthetic groundwater was rapid, confirming the viability of the Hanford sediments used in these experiments. Resazurin added to the synthetic groundwater was reduced from blue to pink in all of the biologically active experimental microcosms within 5 days of static incubation (28°C, dark), while the dye remained oxidized (blue) in the abiotic (heat killed) and baseline (no groundwater added) controls for the duration of the experiment (40 days). While qualitative, these results demonstrate the ability of the soil microbiome to quickly activate respiration in response to soil wetting. Microbial cell densities were estimated from original sediments by QPCR to be 2.24 × 10^7^ (± 0.1, standard deviation) 16S rRNA gene equivalents/gram for the no ^129^I containing ZP-1 sediment, 1.27 × 10^6^ (± 0.05 SD) 16S rRNA gene equivalents/gram for the low ^129^I (1.0 μg/L ^129^I) UP-1 sediment, and 2.79 × 10^5^ (± 0.1 SD) 16S rRNA gene equivalents/gram for the high ^129^I (10 μg/L ^129^I) UP-1 sediment (Table 1).

Methyl-iodide was produced by nearly all (52 out of 54) of the sediment microcosms that were spiked with a I^−^ (from KI) stock solution, regardless of prior exposure to radioiodine contamination or the experimental treatments conducted in the laboratory. No other gaseous iodine compounds were detected. Only two microcosms, both derived from no ^129^I containing ZP-1 sediments but different treatments, failed to produce methyl-iodide by the end of the 40-day incubation period. Methyl-iodide was not detected in the heat-killed abiotic control sediments, suggesting that methyl-iodide was being produced microbiologically. This unanimous result is consistent with numerous studies that have shown that iodine volatilizing bacteria are ubiquitous in soils and sedimentary environments (Amachi et al., 2001; Yeager et al., 2017), but the yields reported here are considerably higher than previous reports for natural environments. Overall, methyl-iodide yields did not differ significantly (*t*-test, *p* > 0.05) between sediments based on prior exposure to ^129^I or experimental concentration of KI^−^ (Table 2). These results are inconsistent with previous reports that have shown a positive correlation between iodine volatilization and concentration (e.g., Yoshida and Muramatsu, 1995; Redeker et al., 2000; Amachi et al., 2001; Dimmer et al., 2001). The source of these inconsistencies could be a system-specific feature(s) that differs from shallow terrestrial and marine soils, a response to the low concentrations of spiked iodide used here relative to other published studies, or possibly due to interference by background ^127^I in these sediments. Considerable levels of ^127^I were co-disposed with ^129^I to the subsurface at the Hanford Site. Only ^129^I values were reported for these sediments, no data were available for ^127^I. Elevated concentrations of total iodine (^127^I and ^129^I) in the samples could help explain the high production yields of methyl-iodide across all three sediments; reinforcing the hypothesis that iodine contamination primes the subsurface microbial community for iodine methylation and volatilization. Additionally, background ^127^I would also contribute to the high transformation efficiencies calculated in Table 3. These studies do not provide unequivocal evidence that iodine volatilization is occurring in the Hanford subsurface, but an averaged experimental conversion of >60% of the total iodide mass to methyl-iodide underscores a tremendous potential that could be significant depending on the temporal and spatial scales of activity *in situ*.

**Table 2:** Averaged (± standard deviation) iodomethane (nmol/L) production by sediment core and experimental iodide concentration.

Core	KI^−^ (μg/L)	Iodomethane (nmol/L)	Range (nmol/L)	% Total
ZP-1, No ^129^I		95.96 ± 73.21	187.06–1.42	43.46 ± 33.89
UP-1, 1 μg/L ^129^I		193.33 ± 44.52	264.86–76.24	88.56 ± 31.81
UP-1, 10 μg/L ^129^I		150.21 ± 82.04	264.51–51.31	67.99 ± 43.87
	150	160.31 ± 81.87	264.51–33.37	91.55 ± 46.76
	200	149.44 ± 77.39	240.36–1.42	64.01 ± 33.15
	250	129.74 ± 76.89	264.86–1.42	44.45 ± 26.35

*The total % iodine volatilized calculation was based only on the iodide added experimentally. Background iodine (^**129**^I, ^**127**^I) in the sample was not taken into consideration*.

**Table 3:** Averaged (± standard deviation) iodomethane (ug/L) production on a cellular basis by site and treatment.

Core	KI^−^ (μg/L)	OC	% Total I volatilized	fmol CH_3_I/cell (± SD, *n* = 3)
ZP-1, No ^129^I				0.48 (± 0.37)
	150	+	23.65 ± 4.3	0.21 (± 0.04)
	200	+	15.68 ± 17.11	0.18 (± 0.2)
	250	+	1.67 ± 1.11	0.02 (± 0.02)
	150	−	94.55 ± 1.81	0.83 (± 0.02)
	200	−	65.27 ± 4.9	0.77 (± 0.06)
	250	−	59.93 ± 4.45	0.88(± 0.07)
UP-1, 1 μg/L ^129^I				0.41 (± 0.09)
	150	+	127.06 ± 2.81	0.47 (± 0.01)
	200	+	80.5 ± 5.24	0.4 (± 0.03)
	250	+	51.76 ± 34.32	0.32 (± 0.2)
	150	−	120.28 ± 2.87	0.45 (± 0.01)
	200	−	94.4 ± 1.35	0.47 (± 0.0070.1)
	250	−	57.37 ± 2.49	0.36 (± 0.02)
UP-1, 10 μg/L ^129^I				1.45 (± 0.8)
	150	+	39.15 ± 10.47	0.66 (± 0.2)
	200	+	29.27 ± 6.35	0.66 (± 0.14)
	250	+	27.92 ± 3.66	0.79 (± 0.1)
	150	−	144.61 ± 5.71	2.45 (± 0.1)
	200	−	98.92 ± 3.75	2.24 (± 0.1)
	250	−	68.07 ± 0.39	1.92 (± 0.01)

*The total % iodine volatilized calculation was based on the amount of iodide added experimentally. Background iodine (^**129**^I, ^**127**^I) in the sample was not taken into consideration*.

**Figure 1:**
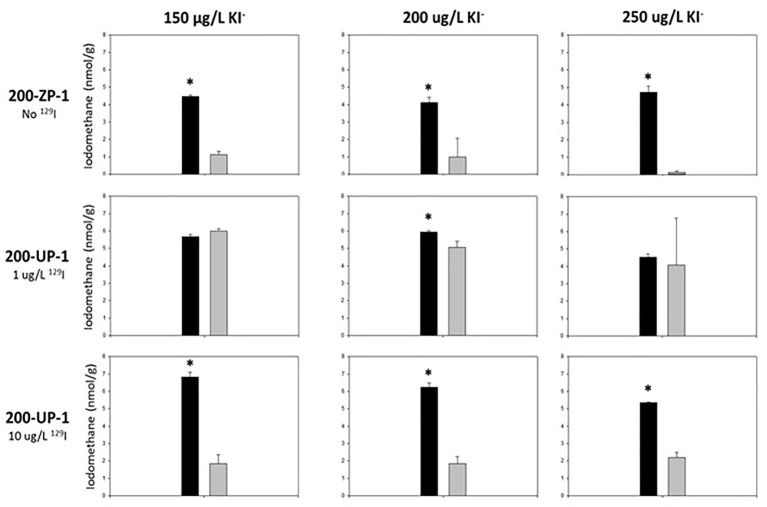
Iodomethane production from Hanford subsurface sediments. Bar graphs show averaged (± standard deviation) iodomethane production among experimental triplicates. Black bars indicate sediment microcosms that only received synthetic groundwater. Gray bars indicate sediment microcosms that received synthetic groundwater, glucose (1 mM), and yeast extract (20 g/L). Student *t*-tests were used to determine statistical significance (*p* < 0.05, indicated by “*”) in iodomethane production between treatments.

Surprisingly, methyl-iodide yields were significantly higher under native (oligotrophic) Hanford sediment conditions (Table 4; Figure 1), indicating that iodide methylation and volatilization activity did not require nutrient supplementation for activation or stimulation. Methyl-iodide yields were consistently higher in 200-ZP-1 (No ^129^I) and UP-1 (High ^129^I) microcosms that did not receive glucose and yeast extract supplementation compared to those that did (Table 4; Student’s *t*-test *p* < 0.05). By contrast, methyl-iodide production in the 200-UP-1 (Low ^129^I) microcosms was less responsive to nutrient addition; activity was significantly suppressed for only one of the I^−^ concentration treatments (Figure 1). These results diverge from published studies on rice paddy soils where glucose and yeast extract supplementation stimulated biological emission of methyl-iodide (Amachi et al., 2003). The suppression of methylation activity may provide some clues about the microbes that are contributing to iodine emission in Hanford sediments. Many studies have demonstrated the ability of diverse terrestrial bacteria and fungi to methylate iodide (Harper, 1985; Amachi et al., 2001, 2003; Redeker et al., 2004; Duborská et al., 2017). While these studies were not exhaustive and strain-specific differences are apparent; we can draw the generalization that methyl-iodide formation by bacteria is stimulated by the availability of a carbon substrate, while production is suppressed in fungi (Harper, 1985; Amachi et al., 2001, 2003; Redeker et al., 2004; Duborská et al., 2017). Applying this pattern to the results for the ZP-1 (No ^129^I) and UP-1 (Hi, 10 μg/L ^129^I) microcosms, we would hypothesize that fungi could play an important role in iodide methylation in these subsurface sediments (Figure 1). The results are less straightforward for the UP-1 (Lo 1 ug/L ^129^I) microcosms, and may reflect a more dynamic microbial community response (e.g., Amachi et al., 2001, 2003). More detailed investigations are needed to decipher the relative contributions of bacteria and fungi to iodide volatilization in this system, as well as to characterize the geochemical conditions that coincide with methyl-iodide formation.

**Table 4:** Averaged (± standard deviation) iodomethane (nmol/L) production by experimental treatment.

	ZP-1, No ^129^I	UP-1, 1 μg/L ^129^I	UP-1, 10 μg/L ^129^I
Baseline (No additions)	164.28 ± 13.17*	199.49 ± 24.85	227.62 ± 24.68*
Glucose (1 mM), YE (1 g/L)	27.63 ± 26.67	187.17 ± 59.24	72.80 ± 14.50
Heat-killed, abiotic controls	0.0 ± 0.0	0.0 ± 0.0	0.0 ± 0.0

Student’s *t*-test was used to test for statistical significance (*p* < 0.05), and is indicated by “*”.

Keppler et al. (2000) demonstrated that abiotic reaction mechanisms in sediments can be a significant source of halogenated alkanes, including methyl-iodide. Here, no methyl-iodide was detected from microcosms prepared with heat-killed sediments, confirming microbiological methylation of iodide. These controls do not discount the possibility of abiotic reactions completely, though, because heat inactivation could alter mineral structures and, potentially, the reactivity of the sediments. The proposed mechanism for abiotic methylation couples the oxidation of natural organic matter to a redox metal mediator for the methylation of iodide (Keppler et al., 2003; Allard et al., 2010). It is noteworthy that relatively high concentrations of reactants (mM levels of Fe, I^−^ and > 6% OC) were required to produce pM quantities of iodo-alkanes experimentally. Not only would these conditions be highly atypical in deep subsurface Hanford sediments (sub-μM levels of Fe, I^−^ and < 0.1% OC) but the basic pH of the system does not favor abiotic methylation reactions. The yields we report for the biotic formation of methyl-iodide are three orders of magnitude higher than those reported for abiotic processes under ideal conditions (Keppler et al., 2003); we therefore conclude that abiotic pathways for the production of methyl-iodide are most likely insignificant sources in the Hanford subsurface.

To the best of our knowledge, this is the first investigation to unequivocally demonstrate the methylation and volatilization of iodide by deep terrestrial subsurface microbial communities inhabiting radioiodine-contaminated sediments at a nuclear waste site. This study does not provide *in situ* rates of methyl-iodide production, but clearly demonstrates the iodide methylation could have an important influence on the fate and distribution of iodine in the Hanford subsurface. We would anticipate that iodide methylation activity would be most prevalent at the capillary fringe where a supply of iodide and oxygenated groundwater converge in sediments that harbor a high standing stock of viable microbial biomass capable of emitting methyl-iodide. Biotic volatilization of iodide from the groundwater (liquid phase) to the vadose zone (gas phase) could have important implications for the remediation and long-term stewardship of ^129^I impacted sites, but more detailed investigations are needed to quantify the spatiotemporal magnitude of this pathway *in situ*.

## Data Availability Statement

All datasets generated for this study are included in the article.

## Author Contributions

CB, LZ, JW, AM, and NQ contributed intellectual input and assistance to this study and manuscript preparation. CB developed the original framework. LZ, JW, and AM contributed reagents, analytical support and data analysis. CB performed statistical analysis and data integration. CB wrote the paper.

### Conflict of Interest

The authors declare that the research was conducted in the absence of any commercial or financial relationships that could be construed as a potential conflict of interest.

## References

[ref1] Allard S. Gallard H. Fontaine C. Croué J.-P. (2010). Formation of methyl iodide on a natural manganese oxide. Water Res. 44, 4623–4629. 10.1016/j.watres.2010.06.008, PMID: 20580399

[ref2] Amachi S. (2008). Microbial contribution to global iodine cycling: volatilization, accumulation, reduction, oxidation, and sorption of iodine. Microbes Environ. 23, 269–276. 10.1264/jsme2.ME08548, PMID: 21558718

[ref3] Amachi S. Kamagata Y. Kanagawa T. Muramatsu Y. (2001). Bacteria mediate methylation of iodine in marine and terrestrial environments. Appl. Environ. Microbiol. 67, 2718–2722. 10.1128/AEM.67.6.2718-2722.2001, PMID: 11375186 PMC92930

[ref4] Amachi S. Kasahara M. Hanada S. Kamagata Y. Shinoyama H. Fujii T.. (2003). Microbial participation in iodine volatilization from soils. Environ. Sci. Technol. 37, 3885–3890. 10.1021/es0210751, PMID: 12967109

[ref5] Dimmer C. H. Simmonds P. G. Nickless G. Bassford M. R. (2001). Biogenic fluxes of halomethanes from Irish peatland ecosystems. Atmos. Environ. 35, 321–330. 10.1016/S1352-2310(00)00151-5

[ref6] Duborská E. Urík M. Bujdoš M. (2017). Comparison of iodide and iodate accumulation and volatilization by filamentous fungi during static cultivation. Water Air Soil Pollut. 228, 225. 10.1007/s11270-017-3407-4

[ref7] Fredrickson J. K. Zachara J. M. Kennedy D. W. Kukkadapu R. K. McKinley J. P. Heald S. M. (2004). Reduction of TcO_4_^−^ by sediment-associated biogenic Fe(II). Geochim. Cosmochim. Acta 68, 3171–3187. 10.1016/j.gca.2003.10.024

[ref8] Fuge R. Johnson C. C. (1986). The geochemistry of iodine. Environ. Geochem. Health 8, 31–54. 10.1007/BF02311063, PMID: 24213950

[ref9] Fuks G. Elgart M. Amir A. Zeisel A. PJ Turnbaugh Y. S. Shental N. (2018). Combining 16S rRNA gene variable regions enables high-resolution microbial community profiling. Microbiome 6:17. 10.1186/s40168-017-0396-x, PMID: 29373999 PMC5787238

[ref10] Gee G. W. Oostrom M. Freshley M. D. Rockhold M. L. Zachara J. M. (2007). Hanford site vadose zone studies: an overview. Vadose Zone J. 6, 899–905. 10.2136/vzj2006.0179

[ref11] Handl J. Oliver E. Jakob D. Johanson K. J. Schuller P. (1993). Biospheric ^129^I concentration in the pre-nuclear and nuclear age. Health Phys. 65, 265–271. 10.1097/00004032-199309000-00003, PMID: 8244695

[ref12] Hao Z. Wang J. Yin Y. Cao D. Liu J. (2018). Abiotic formation of organoiodine compounds by manganese dioxide induced iodination of dissolved organic matter. Environ. Pollut. 236, 672–679. 10.1016/j.envpol.2018.02.001, PMID: 29438953

[ref13] Harper D. B. (1985). Halomethane from halide ion – a highly efficient fungal conversion of environmental significance. Nature 315, 55–57. 10.1038/315055a0

[ref14] He J. Ritalahti K. M. Aiello M. R. Löffler F. E. (2003). Complete detoxification of vinyl chloride by an anaerobic enrichment culture and identification of the reductively dechlorinating population as a *Dehalococcoides* species. Appl. Environ. Microbiol. 69, 996–1003. 10.1128/AEM.69.2.996-1003.2003, PMID: 12571022 PMC143667

[ref15] Hou X. L. Fogh C. L. Kucera J. Andersson K. G. Dahlgaard H. Nielsen S. P. (2003). Iodine-129 and caesium-137 in Chernobyl contaminated soil and their chemical fractionation. Sci. Total Environ. 308, 97–109. 10.1016/S0048-9697(02)00546-6 12738204

[ref16] Hou X. Hou Y. (2012). Analysis of 129I and its application as environmental tracer. J. Anal. Sci. Technol. 3, 135–153. 10.5355/JAST.2012.135

[ref17] Hu Q. Zhao P. Moran J. E. Seaman J. C. (2005). Sorption and transport of iodine species in sediments from the Savannah River and Hanford sites. J. Contam. Hydrology 78, 185–205. 10.1016/j.jconhyd.2005.05.007 16019109

[ref18] Kaplan D. I. Denham M. E. Zhang S. Yeager C. Xu C. Schwehr K. A.. (2014). Radioiodine biogeochemistry and prevalence in groundwater. Crit. Rev. Environ. Sci. Technol. 44, 2287–2335. 10.1080/10643389.2013.828273, PMID: 25264421 PMC4160254

[ref19] Keppler F. Borchers R. Elsner P. Fahimi I. Pracht J. Schöler H. F. (2003). Formation of volatile iodinated alkanes in soil results from laboratory studies. Chemosphere 52, 477–483. 10.1016/S0045-6535(03)00198-X, PMID: 12738273

[ref502] Keppler F. Eiden R. Niedan V. Pracht J. Schöler H. F. (2000). Halocarbons produced by natural oxidation processes during degradation of organic matter. Nature 403, 298–301. 10.1038/35002055, PMID: 10659846

[ref20] Kerisit S. N. Smith F. N. Hoover M. E. Saslow S. A. Qafoku N. (2018). Incorporation modes of iodate in calcite. Environ. Sci. Technol. 52, 5902–5910. 10.1021/acs.est.8b00339 29699395

[ref21] Lawter A. R. Garcia W. L. Kukkadapu R. K. Qafoku O. Bowden M. E. Saslow S. A.. (2018). Technetium and iodine aqueous species immobilization and transformations in the presence of strong reductants and calcite-forming solutions: remedial action implications. Sci. Total Environ. 636, 588–595. 10.1016/j.scitotenv.2018.04.240, PMID: 29723831

[ref22] Li J. Zhou H. Wang Y. Xie X. Qian K. (2017). Sorption and speciation of iodine in groundwater system: the roles of organic matter and organic-mineral complexes. J. Contam. Hydrol. 201, 39–47. 10.1016/j.jconhyd.2017.04.008 28495233

[ref23] Manley S. L. (2002). Phytogenesis of halomethanes: a product of selection or a metabolic accident? Biogeochemistry 60, 163–180. 10.1023/A:1019859922489

[ref25] Moore R. M. Groszko W. (1999). Methyl iodide distribution in the ocean and fluxes to the atmosphere. J. Geophys. Res. Oceans 104, 11163–11171. 10.1029/1998JC900073

[ref26] Muramatsu Y. Ohmomo Y. (1986). Iodine-129 and iodine-127 in environmental samples collected from Tokaimura/Ibaraki, Japan. Sci. Total Environ. 48, 33–43. 10.1016/0048-9697(87)90207-5 3438739

[ref27] Rao U. Fehn U. Muramatsu Y. McNeil H. Sharma P. Elmore D. (2002). Tracing the history of nuclear releases: determination of ^129^I in tree rings. Environ. Sci. Technol. 36, 1271–1275. 10.1021/es011045i, PMID: 11944679

[ref28] Redeker K. R. Treseder K. K. Allen M. F. (2004). Ectomycorrhizal fungi: a new source of atmospheric methyl halides. Glob. Chang. Biol. 10, 1009–1016. 10.1111/j.1529-8817.2003.00782.x

[ref29] Redeker K. R. Wang N. JC Low A. M. M. Tyler S. C. Cicerone R. J. (2000). Emissions of methyl halides and methane from rice paddies. Science 290, 966–969. 10.1126/science.290.5493.966, PMID: 11062125

[ref500] Santschi P. H. Schwehr K. A. (2004). I-129/I-127 as a new environmental tracer or geochronometer for biogeochemical or hydrodynamic processes in the hydrosphere and geosphere: The central role of organo-iodine. Sci. Total. Environ. 321, 257–271. 10.1016/j.scitotenv.2003.09.003 15050400

[ref501] Schmitz K. Aumann D. C. (1995). A study on the association of two iodine isotopes of natural I-127 and of the fission product I-129, with soil components using a sequential extraction procedure. J. Radioanal. Nucl. Chem. 198, 229–236.

[ref30] Whitehead D. C. (1984). The distribution and transformations of iodine in the environment. Environ. Int. 10, 321–339. 10.1016/0160-4120(84)90139-9

[ref31] Xu C. Kaplan D. I. Zhang S. Athon M. Ho Y.-F. Li H.-P. (2015). Radioiodine sorption/desorption and speciation transformation by subsurface sediments from the Hanford site. J. Environ. Radioact. 139, 43–55. 10.1016/j.jenvrad.2014.09.012 25464040

[ref32] Xu C. Miller E. J. Zhang S. J. Li H. P. Ho Y. F. Schwehr K. A.. (2011). Sequestration and remobilization of radioiodine (^129^I) by soil organic matter and possible consequences of the remedial action at Savannah River site. Environ. Sci. Technol. 45, 9975–9983. 10.1021/es201343d, PMID: 22035296

[ref33] Yeager C. M. Amachi S. Grandbois R. DI Kaplan C. X. Schwehr K. Santschi P. H. (2017). Microbial transformation of iodine: from radioisotopes to iodine deficiency. Adv. Appl. Microbiol. 101, 83–136. 10.1016/bs.aambs.2017.07.002 29050668

[ref34] Yoshida S. Muramatsu Y. (1995). Determination of organic, inorganic, and particulate iodine in the coastal atmosphere of Japan. J. Radioanal. Nucl. Chem. 196, 295–302.

[ref35] Zachara J. Serne J. Freshley M. Mann F. Anderson F. Wood M. (2007). Geochemical processes controlling migration of tank wastes in Hanford’s vadose zone. Vadose Zone J. 6, 985–1003. 10.2136/vzj2006.0180

[ref36] Zhang S. Xu C. Creeley D. Ho Y.-F. Li H.-P. Grandbois R.. (2013). Iodine-129 and Iodine-127 speciation in groundwater at the Hanford site, U.S.: iodate incorporation into calcite. Environ. Sci. Technol. 47, 9635–9642. 10.1021/es401816e, PMID: 23885783

